# Propensity Score-Matched Analysis on the Association Between Pregnancy Infections and Adverse Birth Outcomes in Rural Northwestern China

**DOI:** 10.1038/s41598-018-23306-5

**Published:** 2018-03-26

**Authors:** Leqian Guo, Pengfei Qu, Ruo Zhang, Doudou Zhao, Hongli Wang, Rong Liu, Baibing Mi, Hong Yan, Shaonong Dang

**Affiliations:** 1 Department of Epidemiology and Biostatistics, School of Public Health, Xi’an Jiaotong University Health Science Center, Xi’an, Shaanxi 710061 China; 2 Assisted Reproduction Center, Northwest Women’s and Children’s Hospital of Xi’an Jiaotong University, Xi’an, Shaanxi 710003 China; 3 Translational Medicine Center, Northwest Women’s and Children’s Hospital of Xi’an Jiaotong University, Xi’an, Shaanxi 710061 China

## Abstract

The purpose of this study is to examine the relationship between infections and birth outcomes in pregnant Chinese women by using propensity score (PS) matching. The data used here was from a large population-based cross-sectional epidemiological survey on birth defects in Shaanxi province, Northwest China. The babies born during 2010–2013 and their mothers were selected with a stratified multistage sampling method. We used PS-matched (1:1) analysis to match participants with infections to participants without infections. Of 22916 rural participants, the overall prevalence of infection was about 39.96%. 5381 pairs were matched. We observed increased risks of birth defects with infections, respiratory infections and genitourinary infections during the pregnancy (OR, 1.59; 95% CI: 1.21–2.08; OR, 1.44; 95% CI: 1.10–1.87; OR, 3.11; 95% CI: 1.75–5.54). There was also a significant increase of low birth weight associated with respiratory infections (1.13(1.01–1.27)). The association of birth defect with the infection could be relatively stable but the effect could be mediated by some important factors such as mother’s age, education level and economic level. The infection during pregnancy is common in Chinese women and might increase the risk of offspring birth defects and low birth weight, especially in younger, lower education, poor pregnant women.

## Introduction

Maternal and child health is a part of the most important global public health problems, which has been included as a notable part of United Nations Millennium Development Goals^1^. As a widely existing health threat, adverse birth outcomes such as birth defects, low birth weight (LBW) and preterm birth (PTB) can produce great disease burden on fetal health status at birth, as well as potential developmental problems in childhood and future the risk of various diseases^2–4^. For example, birth defects, also known as the congenital disorder, are a condition existing at or before birth regardless of cause. They are the primary causes of infant mortality, and different disabilities are in developed countries. Congenital anomalies resulted in about 632,000 deaths per year in 2013 down from 751,000 in 1990^5^. Birth defects are present in about 3% of newborns in the USA^6^. In China, CHD is the most prevalent birth defect, accounting for over 10% of cases of infant mortality in 2008^7^. As for another adverse birth outcome, LBW (<2500 g) also contributes a lot to infant mortality and childhood morbidity, as well as individual health during the life course^8^. More than 20 million infants worldwide, accounting for 15.5% of all births, are born with LBW, and 95.6% of these babies occurred in developing countries^9^, especially in rural areas. Preterm birth (<37 weeks of gestation) is the main cause of neonatal death and the second leading cause (after pneumonia) in children under 5 years of age^10^. The estimated number of PTB infants was approximately 15 million in 2010 worldwide, which represented 11% of all live births^11^.

The mechanisms of adverse birth outcomes are incompletely understood. Throughout gestation, they are generally considered induced by the complex effects of genetic and multiple environmental risk factors^12^. For instance, lack of maternal multivitamin use^13^, maternal age, education and economic level, maternal use of drug^13^, alcohol^14^, and cigarettes^15^ during pregnancy have all been associated with the occurrence of fetal anomalies.

There is also growing evidence that some infections during pregnancy may play an important role in the occurrence of adverse pregnancy outcomes and congenital anomalies^16–18^. Infections are the result of infectious agents including viruses, viroids, prions, bacteria, nematodes and so on. For instance, pregnant women are vulnerable to viral respiratory infections^19^ and are found to be at high risk for influenza and related complications^17,20^. Influenza during pregnancy has been linked to spontaneous abortion, stillbirth, and prematurity^21,22^. Maternal fever is also common during pregnancy. Botto *et al*. estimated that 5–10% of women, on average, report a fever in early pregnancy^23^. It was reported that fever has been suspected to harm the developing fetus^24^. Even short exposures to elevated maternal body temperature have been reported to lead to cell death, vascular disruptions, and placental infarction^25^. In addition, urinary tract infections, especially pyelonephritis^26^ are reported to associate with preterm birth.

However, these studies have a few limitations. Prospective cohort studies with laboratory confirmation of infections are ideal but are often hampered by low numbers of events, particularly for outcomes such as fetal birth defects. Existing birth registries can facilitate large studies, but they do not routinely collect other more confounders. In other words, a number of study design issues have limited the interpretation of previous results because of small sample sizes and lack of assessment of potential confounders. Moreover, individuals with mild or subclinical infections may never seek medical care. There will have a lower infection rate.

An observational study, in contrast, is often limited by potential residual biases from measured confounders and possible biases due to unmeasured confounders^27^. The propensity score (PS) technique has turned out to be associated with the largest reduction selection bias and residual biases assessing causal effects in observational studies^28,29^. PS matching estimates the probability of individuals being “exposed” to infections and allows comparison with “unexposed” people with similar demographic and socioeconomic characteristics, that is, with a similar profile of potential confounders. Using a PS approach to estimate differences in adverse birth outcomes between groups of people with and without infections is an important alternative method to “standard” multivariable regression adjustment to estimate the unbiased association of infections with birth outcomes, and hence the causal association in the absence of confounding, random error, measurement error, and selection biases. In addition, in sparsely populated Northwestern China, economic condition is worse and health services are less developed than in Eastern and Coastal areas of China. Consequently, this large population-based observational study has been carried out to better understand, by using PS methods, the potential effects of exposure to maternal infections on fetal development and infant health in rural northwestern China. This analysis will be used as a baseline for better quantification and qualification of these risks so as to provide not only research insights into mechanisms of teratogenicity but could be helpful in clinical counseling of exposed women and primary prevention.

## Results

### Characteristics of the participants

22916 participants were available for analysis. The overall prevalence of infections during the pregnancy was 39.96% (9157/22916). Of these, the prevalence of respiratory infections and genitourinary infections were 38.99% and 1.82%, respectively. After propensity score matching, 5381 participants with infection were matched one to one to participants without infection. The overall prevalence of adverse birth outcomes was 3444 participants (15.41%). Of these, birth defects were 486 participants (2.12%), LBW was 2773 participants (12.41%) and PTB was 613 participants (2.68%).

**Figure 1:**
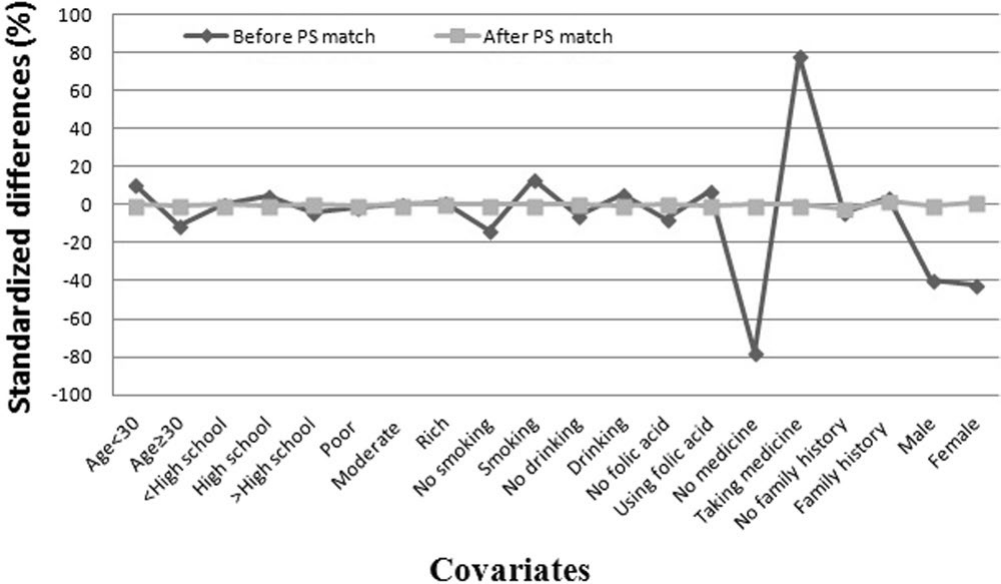
Standardized differences before and after PS matching comparing covariate values for participants having and not having infections.

Before PS-matching, participants’ characteristics varied considerably among the two groups (Table 1). We found the proportion of maternal age (≥30), education (<High school) in the group without infection was higher than that in the group with infection (23.57% vs 19.13%, 72.86% vs 71.04%). Participants with infection had a greater proportion of maternal passive smoking, drinking, folic acid supplement use, taking medicine and family history of birth defects. In addition, the wealth index and baby gender between the groups had no significant difference among participants. After PS-matching, the 5381 matched pairs were analyzed for differences in the baseline characteristics and outcome variables (Table 1). There were no significant differences between the two matched groups. Across 9 covariates, the standardized differences were between −1.80 and 1.80, with a reduction of 5% demonstrating that all variables were sufficiently balanced between the two matched groups (Fig. 1).

**Table 1:** Baseline characteristics by infection before and after propensity score (PS) matching.

Covariate	Before PS match			After PS match		
	no infection	infection	*p*-value	no infection	infection	*p*-value
Maternal age						
<30	10353(76.43)	7316(80.87)	<0.001	4407(81.90)	4408(81.92)	0.980
≥30	3193(23.57)	1731(19.13)		974(18.10)	973(18.08)	
Maternal education						
<High school	9999(72.86)	6494(71.04)	0.003	3854(71.62)	3861(71.75)	0.986
High school	2600(18.95)	1898(20.76)		1080(20.07)	1073(19.94)	
>High school	1124(8.19)	749(8.19)		447(8.31)	447(8.31)	
Wealth index						
Poor	3180(28.87)	2147(28.22)	0.539	1506(27.99)	1490(27.69)	0.911
Moderate	5476(49.72)	3795(49.88)		2611(48.52)	2611(48.52)	
Rich	2357(21.4)	1667(21.91)		1264(23.49)	1280(23.79)	
Maternal passive smoking						
No	10404(75.88)	6382(69.97)	<0.001	3786(70.36)	3786(70.36)	>0.999
Yes	3307(24.12)	2739(30.03)		1595(29.64)	1595(29.64)	
Maternal drinking						
No	13613(99.08)	9000(98.48)	<0.001	5343(99.29)	5344(99.31)	0.908
Yes	127(0.92)	139(1.52)		38(0.71)	37((0.69)	
Maternal folic acid supplement use						
No	6614(48.1)	4065(44.39)	<0.001	2423((45.03)	2437(45.29)	0.786
Yes	7136(51.9)	5092(55.61)		2958((54.97)	2944(54.71)	
Maternal taking medicine						
No	13055(94.92)	6048(66.07)	<0.001	4836(89.87)	4836(89.87)	>0.999
Yes	699(5.08)	3106(33.93)		545(10.13)	545(10.13)	
Family history of birth defects						
No	13664(99.31)	9061(98.95)	0.004	5356(99.54)	5349(99.41)	0.353
Yes	95(0.69)	96(1.05)		25(0.46)	32(0.59)	
Baby gender						
Male	7554(59.74)	5090(40.26)	0.311	3021(56.14)	2997(55.70)	0.641
Female	6201(60.4)	4065(39.6)		2360(43.86)	2384(44.30)

### Infections and adverse birth outcomes

Table 2 showed the results of the logistic regression and PS-matched analyses between infections and adverse birth outcomes. Prior to matching, the risk of birth defects was significantly greater among the participants with infections, adjusting for the confounders in Table 2 [odds ratio (OR), 1.55; 95% confidence interval (CI), 1.23–1.93]. Similarly, the risks of birth defects were significantly greater among the participants with respiratory infections and genitourinary infections during the pregnancy (OR, 1.41; 95% CI: 1.13–1.77; OR, 2.43; 95% CI: 1.51–3.89). After PS-matching, compared with before PS-matching, the association became stronger. The ORs for birth defects were 1.59(95% CI: 1.21–2.08), 1.44(95% CI: 1.10–1.87), 3.11(95% CI: 1.75–5.44), respectively. There was also a significant increase of low birth weight associated with respiratory infections (OR, 1.13; 95% CI: 1.01–1.27). There was no statistical significance between infections and PTB.

**Table 2:** Odds ratios and 95% CI for infections and adverse birth outcomes (OR(95% CI)).

Infections	Birth outcomes	Before PS-matching		After PS-matching
		Unadjusted	Adjusted^a^	
Overall infections	Birth defects	1.97(1.64–2.36)	1.55(1.23–1.93)	1.59(1.21–2.08)
	Low birth weight	1.06(0.98–1.15)	1.11(1.01–1.23)	1.11(0.99–1.25)
	Preterm birth	1.01(0.86–1.19)	1.09(0.89–1.34)	1.02(0.80–1.31)
Respiratory infections	Birth defects	1.86(1.55–2.22)	1.41(1.13–1.77)	1.44(1.10–1.87)
	Low birth weight	1.07(0.98–1.16)	1.12(1.01–1.24)	1.13(1.01–1.27)
	Preterm birth	1.02(0.87–1.21)	1.11(0.90–1.36)	1.01(0.79–1.28)
Genitourinary infections	Birth defects	2.77(1.80–4.26)	2.43(1.51–3.89)	3.11(1.75–5.54)
	Low birth weight	1.16(0.85–1.59)	1.14(0.81–1.62)	1.05(0.70–1.58)
	Preterm birth	1.66(1.02–2.67)	1.54(0.90–2.62)	1.77(0.90–3.50)

^a^Adjusted maternal age, maternal education, socioeconomic status, maternal passive smoking, drinking, folic acid supplement use, taking medicine, family history of birth defects and baby gender.

### Subgroup analyses

Table 3 presented the outcomes of subgroup analyses after PS matching. Apart from pregnant women taking medication during the pregnancy, all effects of subgroups were on the similar side of the forest plot, with a positive effect of infections on birth defects (OR > 1), although some effects were not statistically significant. Some significantly associations were also found such as in the subgroups of maternal age (<30) (OR, 1.71; 95% CI: 1.25–2.33), maternal education (<high school) (OR, 1.52; 95% CI: 1.12–2.07), poor (OR, 2.23; 95% CI: 1.38–3.60), and female baby (OR, 2.49; 95% CI: 1.53–4.05).There are higher risks in these subgroups.

**Table 3:** The forest plot of association between infection and birth defects among different subgroups after PS match.

covariate	no infection	infection		*OR(95%CI)*	*P*-value
	n = 5381	n = 5381			
Maternal age					
<30	65(1.47)	110(2.50)		1.71(1.25–2.33)	0.001
≥30	24(2.46)	30(3.08)		1.26(0.73–2.17)	0.406
Maternal education					
>High school	3(0.67)	8(1.79)		2.70(0.71–10.23)	0.145
High school	17(1.57)	28(2.61)		1.68(0.91–3.08)	0.097
<High school	69(1.79)	104(2.69)		1.52(1.12–2.07)	0.008
Wealth index					
Poor	25(1.66)	54(3.62)		2.23(1.38–3.6)	0.001
Moderate	43(1.65)	64(2.45)		1.50(1.02–2.22)	0.042
Rich	21(1.66)	22(1.72)		1.04(0.57–1.89)	0.911
Maternal passive smoking					
No	60(1.58)	79(2.09)		1.32(0.94–1.86)	0.105
Yes	29(1.82)	61(3.82)		2.15(1.37–3.36)	0.001
Maternal drinking					
No	88(1.65)	138(2.58)		1.58(1.21–2.07)	0.001
Yes	1(2.63)	2(5.41)		2.11(0.18–24.37)	0.548
Maternal folic acid supplement use					
No	38(1.57)	66(2.71)		1.75(1.17–2.61)	0.007
Yes	51(1.72)	74(2.51)		1.47(1.02–2.11)	0.036
Maternal taking medicine					
No	67(1.39)	121(2.5)		1.83(1.35–2.47)	<0.001
Yes	22(4.04)	19(3.49)		0.86(0.46–1.61)	0.633
Family history of birth defects					
No	87(1.62)	134(2.51)		1.56(1.18–2.04)	0.001
Yes	2(8.00)	6(18.75)		2.65(0.49–14.47)	0.259
Baby gender					
Male	66(2.18)	83(2.77)		1.28(0.92–1.77)	0.145
Female	23(0.97)	57(2.39)		2.49(1.53–4.05)	<0.001

## Discussion

### Main findings

In the present study, there were relatively high prevalences of adverse birth outcomes and high risk of being infection during pregnancy. Our findings demonstrated that the infection during the pregnancy was associated with increased risk of birth defects and low birth weight. There was also an increased risk of infections and preterm birth but remained not significant statistically. In addition, the association of birth defect with the infection could be stable relatively but the effect of the infection could be mediated by some important factors such as mother’s age, education level, economic level and gender of the baby. These findings are important to strengthen the guidance of pregnancy and reduce the incidence of adverse birth outcomes.

### Comparison with previous studies

Our finding was supported by several studies that found significant associations between congenital defects and febrile illness caused by infections^13,30,31^. Shahrukh *et al*. observed a modestly increased risk (OR, 1.28; 95% CI: 1.01–1.63) evaluating the association between maternal fever and Oral Clefts^13^. Botto *et al*. also found febrile genitourinary infections were associated with selected heart defects, particularly right-sided obstructive defects (OR, >3) and possibly others^23^.

Low birth weight is the result of either preterm birth or poor growth of the fetus resulting in intrauterine growth restriction. After PS-matching, the risk of LBW with respiratory infections increased to 1.13(95% CI: 1.01–1.27) from 1.12(95% CI: 1.01–1.24). Asad *et al*. explored an association between birth weights with the maternal respiratory infection during pregnancy in the community settings^32^, which was similar to our study. Philpott *et al*. also found a significant effect of febrile illness during pregnancy on LBW^33^.

However, we did not observe any strong differences in associations between infections and PTB. This finding contrasted with the general opinion that maternal febrile infections arising during the preterm period can trigger the onset of labor^34,35^. However, there were similar results from other researches, which have not shown an association with preterm birth^36,37^. Erin *et al*. also did not find a significant effect of febrile illness during pregnancy on PTBs^33^. These differences may be the result of inaccuracies in maternal self-report of an infection, which do not always provide with a specific definition. The prevalence of infection could be underestimated or overestimated. In addition, the differences between the region and the population should also be accounted for it.

We further conducted subgroup analysis and found that the association of birth defect with the infection could be stable relatively. However, the effect of the infection could be mediated by some important factors such as mother’s age, education level, economic level, taking medicine during the pregnancy and gender of the baby. Similar to our findings, Shahrukh *et al*.^13^ found an increased risk of oral clefts in women who reported a common cold or influenza and did not take drugs during the illness. In comparison, women who took the medications had no increased risk, which was in complete agreement with our subgroup analysis. Furthermore, hormones, such as progesterone and testosterone, may have regulatory roles in renal development and could potentially influence renal outcomes by gender^30^. Therefore, it is also necessary that the prevention of birth defects should be focused on these pregnant women.

A mechanistically plausible explanation of our findings is that when the infection in pregnancy occurs, the maternal immune system is mobilized, causing changes in the level of cytokines in the fetal environment. Some cytokines, such as tumor necrosis factor-a, interleukin 1 and interleukin 6 are pyrogenic, causing hyperthermia to occur through alteration of the set point in the hypothalamus^38^. Activation of increased body temperature is associated with interruption of protein synthesis and enzyme production, which results in cellular processes (eg, proliferation, migration, differentiation, apoptosis) becoming altered or dysfunctional^38^. Therefore, one mechanism by which maternal hyperthermia might influence fetal development is unregulated apoptosis. Furthermore, it has been suggested that maternal exposure to hyperthermia induces the transcription of protective heat shock proteins, a process that might potentially divert resources from normal protein synthesis away from normal development^39^.

### Strengths and limitations

This study is the first and largest survey that has presently been conducted in Northwest China, and provides up-to-date data on birth defects. In addition, PS-matching method was used to study the relationship between infections and birth defects, LBW, PTB in China, which can reduce confounding as observed covariates are balanced at each particular value of the propensity score^28,29^. After PS-matching, all selected potential confounding factors were sufficiently balanced between the two matched groups in our study. This is analogous to randomization procedures used in clinical trials, as on average the distribution of covariates will be balanced between the infection and non-infection groups, which strengthens causal inference and thus improves the methodological quality of observational research. Compared with conventional matching, PS matching can consider more matching factors and improve the research efficiency. Several limitations should also be addressed while interpreting our results. Firstly, any cause-effect conclusion cannot be drawn due to the limitations of the cross-sectional design, although we used PS-matching to control biases as much as possible. Secondly, the diagnosis of infection may be imprecise due to the self-report and the potential mis-classification of maternal infection occurred. However, the pregnancy is one of important life events for the women and they could care about many events related to pregnancy such as infection during pregnancy. But it might still underestimate the associated effect of infections during pregnancy and adverse birth outcomes because of recall bias. Unfortunately, it is difficult to get laboratory evidence or clinical diagnosis in a large-sample cross-sectional study. Thirdly, we selected whole pregnancy as exposure period rather than a definite stage due to the low incidence of adverse birth defects. Future studies should attempt to consider this effect of differential critical periods using different research design such as case-control study.

## Conclusions

Our findings suggest that the infection during pregnancy in Chinese women was associated with increased risk of birth defects and low birth weight, especially in younger, lower education, poor pregnant women. Therefore, the effective infection control measures in antenatal care should be enhanced further and the high-risk female population should be focused in order to prevent and control adverse birth outcomes.

## Methods

### Data and participants

The data used here was from a large population-based cross-sectional epidemiological survey on birth defects in Shaanxi province, Northwest China between August and December in 2013. The babies born during 2010–2013 and their mothers in this survey were selected with a stratified multistage sampling method. Comprehensive methods of this survey have been previously published elsewhere^40^ and the ethics committee of the Xi’an Jiaotong University Health Science Center approved the study (No. 20120008). All research was performed in accordance with relevant guidelines and regulations and informed consent was obtained from all participants and/or their legal guardians. The datasets analysed during the current study are available from the corresponding author on reasonable request.

All family-level primary data was reported by the mothers in the pre-coded structured family questionnaire, including socio-demographical information, information regarding access to maternal healthcare, health status, and lifestyles during pregnancy, and child-level information such as birth defects and another birth data. Information on birth defects was also collected in the pre-coded structured birth defects questionnaire which contained questions on birth outcomes, diagnostic information at local hospitals, time of diagnosis and the types of defects. Medical records at local hospitals were used as final diagnosis references. In the survey, all questionnaires were designed by Xi’an Jiaotong University Health Science Center. Only after obtaining written informed consent, face-to-face interviews were carried out by the field investigators.

Taking into account the higher percentage of adverse birth outcomes in rural areas, in our present study, we only analyzed the relationship between pregnancy infections and adverse birth outcomes in rural participants. Therefore, the inclusion criterion was a single live birth in rural areas in this article. Excluded were cases of abortion, stillbirth, multiple births, or neonates without the information of having an infection or not. Finally, 22916 singleton live-birth newborns enrolled in this study.

### Outcomes

Previous studies have shown that birth defects, low birth weight (LBW) and preterm birth (PTB) play an impartment role in producing disease burden on fetal health status at birth, as well as potential developmental problems in childhood and future the risk of various diseases^2–4^. Therefore, adverse birth outcomes in this study included these three outcomes. Birth defects were recorded as ICD-10(international classification of diseases) codes and were defined as cleaned malformations, i.e., excluding previously defined insignificant diagnoses^41^. LBW refers to birth weight less than 2500 g and PTB refers to birth at less than 37 weeks gestation. These above outcomes required the definite diagnosis of the hospital.

Data on maternal infections during entire pregnancy were gathered from mother’s self-report or through treatment records if they sought medical care. The reported infections were divided into three types: respiratory infections, genitourinary infections and other infections. Respiratory infections included influenza/common cold (influenza-like symptoms, seasonal influenza), bronchial infections and pneumonia; Genitourinary infections included infections of the kidney, bladder, urinary tract and genital tract; Other infections included gastrointestinal infections and some infections affecting multiple organ systems (e.g., HIV infection, chicken pox) during pregnancy. In brief, maternal infection episode was defined as having any one of the above infections during entire pregnancy.

### Covariates

Based on previous studies^13,30^, such factors as some socio-demographic characteristics, lifestyle, folic acid supplement, taking medicine and family history of birth defects were found associated with adverse birth. So these factors could confound the association between infection and adverse birth outcomes. In our analysis, these factors were controlled. Socio-demographic characteristics captured by the survey included maternal age (<30 and ≥30 years), education (<High school, High school and >High school), socioeconomic status (Poor, Moderate, Rich) and baby gender (male or female). The socioeconomic status was measured using the Demographic and Health Survey wealth index^42^. The principal component analysis was utilized to combine the variables representing families’economic level. On the basis of the first principal component, then, the socioeconomic status of the families was classified into the tertiles representing poor, medium and rich families. Maternal passive smoking and drinking during pregnancy had two dichotomous outcomes variables (no and yes). The folic acid supplement was defined as the number of times when the respondent took folic acid greater than 30 days, and similarly, also classified into two categories (no and yes).

### Statistical Analysis

In the study, the categorical variables were expressed as frequency and percentages and were compared using chi-square tests between the two independent groups. Ordinal data such as education and wealth index were compared using Rank-Sum Test. Logistic regression was performed to investigate the association between infections and birth outcomes. With the purpose of comparing with PS-matching, multivariate logistic regression was also performed before PS-matching. The model included all kinds of birth outcomes as the dependent variable, infections as the exposure, and all the variables mentioned above as potential covariates.

To minimize the possible bias arising from the study design, we constructed a PS model to adjust for potential confounding variables for having an infection or not^28,29^. We performed 1:1 matching by using the calipers matching, which matches each person with and without an infection who has similar propensity scores, within a caliper of 0.0001^43^. The variables selected for the propensity score model included all the covariates above. To evaluate the success of balancing the baseline characteristics between the matched groups, standardized differences were estimated, which directly quantifies the bias in the proportions of covariates across the groups, expressed as a percentage of the pooled standard deviation (SD)^44,45^. The standardized difference was calculated as follows:

If *P*1 and *P*2 represented the prevalence of confounders observed in the exposed and unexposed groups, the percentage standardized difference is:

1 $$100\times \frac{P1-P2}{SD{\rm{poor}}}$$

where

2 $$SD{\rm{poor}}=\sqrt{\frac{P1(1-P1)+P2(1-P2)}{2}}$$

After PS-matching, univariate logistic regressions were performed to explore the association of infections with adverse birth outcomes. Subgroup analyses were conducted further based on selected covariates with the purpose of assessing the robustness of our findings regarding the effect of infections on birth defects and initially identifying high-risk groups.

Data was entered into Epidata 3.1 by double entry (CDC, Atlanta, GA, USA) and all statistical analysis was conducted with STATA version 12.0 software (STATA Corporation, College Station, TX, USA). All statistical tests were evaluated using two-tailed 95% confidence intervals (CI). Statistical significance was set up if P < 0.05.
